# The Treatment of Frontal Sinusitis

**Published:** 1907-10-12

**Authors:** 


### Laryngology and Rhinology.

### THE TREATMENT OF FRONTAL SINUSITIS.

The special features which characterise suppuration within the accessory cavities of the nose, and which were described when we discussed the treatment of antral empyæma a few weeks ago (The Hospital, Sept. 21, p. 663), are well illustrated in disease of the frontal sinus. Thus the cavity, too large to be obliterated by the organised products of inflammation, is enclosed by rigid bony walls and is unable to contract; therefore complete healing can only result by regeneration of the epithelium which lines the healthy sinus or by the surgical removal of so much of the bony wall as will allow of complete obliteration of the cavity. In the latter case the epithelial lining must be thoroughly removed, whereas it should be damaged as little as possible when the former method of cure is intended. The frontal sinus is a cleft between the two tables of the frontal bone; it consists of two portions, a vertical part on the forehead and a horizontal limb extending backwards over the orbit in close relation to the anterior ethmoidal cells. The vertical part is partially subdivided by bony septa and varies very greatly in size. Not infrequently one sinus is much larger than the other, owing to deflection of the partition between them; but at the root of the nose this is always centrally placed, and the infundibulum, or opening from the sinus into the nose, is situated close to it and on the floor of the sinus.

As any external operation on the sinus must necessarily leave some scar on the face, the cosmetic effects of the various methods of treatment are important. Fortunately the nasal opening is placed at the most dependent part of the sinus, and intranasal methods of securing free drainage are often successful in the less severe and more recent cases. The infundibulum should be cleared of all granulations and polypi, and the anterior end of the middle turbinal, which overhangs it, should be removed with scissors and snare. A probe can then be gently passed up to the cavity in a considerable number of cases, and more often when the sinus is diseased than in healthy persons; frequent irrigations by means of a suitable cannula often effect a cure.

Any attempt to forcibly enter the sinus with a trocar or curette must be absolutely condemned as dangerous. These methods frequently fail by reason of narrowness or anatomical variations of the infundibulum, and especially on account of the co-existence of disease of anterior ethmoidal cells which cannot be reached by the intra-nasal route. External operation then becomes necessary, of which the exact method and technique varies greatly with different surgeons. The incision is made in the line of the eyebrow curving inwards and downwards from a point vertically above the inner canthus; the sinus should be opened just above the fronto-nasal suture, for if be present at all it is always to be found at this spot. The opening may be made through the anterior or the inferior wall, but the latter has the balance of advantage for several reasons. The bone is thinner, and, being concave, no depression results, and the ethmoidal cells are more easily reached; but the trochlea of the superior oblique is displaced with the periosteum, though, as it returns to its position at the end of the operation, a slight temporary diplopia in certain positions is usually the most serious result. The opening into the sinus should be large enough to admit the tip of the little finger; the cavity is carefully explored and any septa broken down. A probe is next passed down through the infundibulum, and, guided by a finger in the nose, the anterior ethmoidal cells are very thoroughly removed with a curette or burr; this curettage should be very complete and should be carried backwards through the whole extent of the horizontal limb of the sinus, for persistence of suppuration after operation is most often due to insufficient removal of the ethmoidal cells. The operation may be concluded in various ways; some surgeons pack the cavity with gauze to be removed through the inner part of the wound which is left open, and a second piece of gauze in the nasal opening for removal through the nose; this packing is removed piecemeal, and may be replaced as often as is considered advisable. Other operators prefer a drainage tube passed into the nose, with or without immediate closure of the incision, and some at the present day rely on the large size of the nasal opening made by free removal of the ethmoid and close the incision at once without tube or packing. This latter method is by far the most comfortable for the patient, and gives a rapid convalescence and an almost invisible scar, but in former days a fatal osteomyelitis of the frontal bone has occasionally resulted from retention of pus; however, this can hardly occur if the ethmoidal cells are thoroughly broken down and if care be taken to see that the opening does not become rapidly blocked by polypi.

By such operations provision is made for free drainage of the empyæma, but the discharge will continue for some time and will only entirely cease with the complete regeneration of the epithelial lining. A more rapid and certain cure may be effected by obliteration of the cavity, but this operation leaves considerable deformity, and, when the sinus is large, a huge and most unsightly depression; it should only be performed in the first instance in severe and complicated cases, as where there is necrosis of the walls of the sinus, or in men permission may be obtained to obliterate if the cavity be found to be small; in women, at least, this method should not be performed in ordinary cases until the milder operation has been tried. The first part of the operation is performed, and the ethmoidal region treated in the same way, then the anterior and inferior walls are completely removed and the lining of the cavity carefully dissected away. The depth of the sinus has more influence on the deformity than its superficial extent; the edges of the depression should be carefully levelled, and the dressings so applied as to keep the soft parts in contact with the posterior wall of the sinus; gauze packing may be used for the first few days. Less deformity results if the supra-orbital margin be left as a bony bridge, and some surgeons remove only the anterior wall.

